# Occurrence of *Helicobacter pylori* and Epstein-Barr virus infection in endoscopic and gastric cancer patients from Northern Brazil

**DOI:** 10.1186/1471-230X-14-179

**Published:** 2014-10-15

**Authors:** Carolina Rosal Teixeira de Souza, Kátia Soares de Oliveira, Jefferson José Sodré Ferraz, Mariana Ferreira Leal, Danielle Queiroz Calcagno, Aline Damasceno Seabra, André Salim Khayat, Raquel Carvalho Montenegro, Ana Paula Negreiros Nunes Alves, Paulo Pimentel Assumpção, Marília Cardoso Smith, Rommel Rodríguez Burbano

**Affiliations:** Laboratório de Citogenética Humana, Instituto de Ciências Biológicas, Universidade Federal do Pará, Rua Augusto Corrêa, 01 – Guamá, CEP 66075-110. Caixa postal 479 Belém, PA Brasil; Instituto de Ciências da Saúde, Universidade Federal do Pará, Belém, PA Brasil; Centro Universitário do Pará, Belém, PA Brasil; Departamento de Ortopedia e Traumatologia, Universidade Federal de São Paulo, São Paulo, SP Brazil; Disciplina de Genética, Departamento de Morfologia e Genética, Universidade Federal de São Paulo, São Paulo, SP Brasil; Núcleo de Pesquisa em Oncologia, Universidade Federal do Pará, Belém, PA Brasil; Departamento de Patologia Oral, Faculdade de Odontologia, Universidade Federal do Ceará, Fortaleza, CE Brasil

**Keywords:** *Helicobacter pylori*, *Epstein-Barr virus*, Gastritis, Gastric cancer

## Abstract

**Background:**

*Helicobacter pylori* (HP) and *Epstein-Barr virus* (EBV) have been associated with cancer development. We evaluated the prevalence of HP, HP *CagA*^+^ and EBV infection in gastric cancer (GC) samples from adults and in gastric tissues from patients who underwent upper endoscopy (UE).

**Methods:**

Samples from UE and GC were collected to investigate the presence of HP infection and the HP virulence factor *CagA* by a urease test and PCR. The presence of EBV was detected by *Eber-1 in situ* hybridization.

**Results:**

In UE, 85.5% of juvenile patients showed some degree of gastritis (45.3% of patients with mild gastritis and 54.7% with moderate/severe gastritis) and patients with mild gastritis were younger than patients with moderate/severe gastritis. Among adults, 48.7% presented mild gastritis and 51.3% moderate/severe gastritis. HP infection was detected in 0% of normal mucosa, 58.5% of juvenile gastritis patients, 69.2% of adult gastritis patients and 88% of GC patients. In these same groups, HP *CagA*^*+*^ was detected in 0%, 37.7%, 61.5% and 67.2% of tissue samples, respectively. In juvenile patients, HP infection was more common in those with gastritis than in normal samples (p = 0.004). The patients with either HP or HP *CagA*^*+*^ were older than patients without these pathogens (p < 0.05). In juvenile patients, HP infection was more frequent in cases of moderate/severe gastritis than in cases of mild gastritis (p = 0.026). Moreover, in patients with GC, HP infection was more frequent in males than in females (p = 0.023). GC patients with HP *CagA*^*+*^ were older than patients with HP *CagA*^*-*^ (p = 0.027). HP *CagA*^*+*^ was more common in intestinal-type than diffuse-type GC (p = 0.012). HP *CagA*^*+*^ was also associated with lymph-node (p = 0.024) and distal (p = 0.005) metastasis. No association between EBV infection and HP infection or any clinicopathological variable was detected.

**Conclusions:**

Our results suggest that HP is involved in the pathophysiology of severe gastric lesions and in the development of GC, particularly when *CagA*^*+*^ is present. EBV was not the primary pathogenic factor in our samples.

## Background

Gastric cancer (GC) and other gastrointestinal diseases occur at high rates worldwide [1], and infections involving viruses and bacteria have been associated with these diseases. Recently, several studies have been performed to understand the role of pathogens that infect the human stomach, particularly *Helicobacter pylori* (HP) and *Epstein-Barr virus* (EBV), in gastric carcinogenesis [2–5].

HP, a Gram-negative spiral bacterium, is considered a public health problem. In 1994, the International Agency for Research on Cancer (IARC) defined HP as a group 1 carcinogen [6, 7]. This bacterium colonizes the gastric mucosa of more than 50% of the world’s population [8]. However, only approximately 20% of infected individuals develop severe gastric diseases such as GC. Among the factors- that have been suggested to contribute to development of gastric disease in HP-infected patients are the virulence of HP strains, the permissiveness of the gastric environment and the host genetic background [9]. The HP cytotoxicity associated gene A (*CagA*) is one of the most significant virulence factors of this bacteria, and it has been associated with risk for GC [10].

EBV infects more than 90% of the global adult population, and most individuals are infected during childhood. Upon infection, the virus remains latent in B lymphocytes throughout life [5]. To be oncogenic, EBV must maintain its genome inside host cells to avoid cell death and to evade recognition by the immune system. The contribution of EBV to gastric carcinogenesis has not been fully elucidated [11, 12]. EBV infects epithelial cells from the oropharynx and subsequently spreads to the lymphoid tissues where it infects B lymphocytes [13–15]. Atrophic gastritis may induce the infiltration of EBV-carrying lymphocytes and increase the chance of their contact with the gastric epithelial cells. On the other hand, the gastric inflammation may also produce a cytokine-rich microenvironment to support clonal growth of EBV-infected epithelial cells [16].

In developing countries such as Brazil, HP and EBV infections are particularly prevalent within lower socioeconomic populations. Furthermore, infection occurs at earlier ages in these populations compared to developed countries [17–19]. Studies are needed to determine these pathogens’ association with and influence on the development of gastric diseases at earlier ages, where they could initiate or promote carcinogenic processes. Additionally, the role of HP and EBV in the development of gastric adenocarcinoma in the elderly, the population where this disease is most prevalent, remains unclear.

Therefore, this study aimed to assess the prevalence of HP and EBV infection, as well as the *CagA*-positive status of HP, in gastric tissues from juvenile and adult patients undergoing upper endoscopy (UE) and in tumor specimens from adult patients with GC.

## Methods

### Samples

The present study included: (i) gastric tissue samples from 62 juvenile patients ranging from 12 months to 18 years old, referred for UE to clarify clinical manifestations within the upper gastrointestinal tract, (ii) gastric tissue samples from 39 adult patients ranging from 19 to 61 years old, referred for UE to clarify clinical manifestations within the upper gastrointestinal tract, and (iii) tumor samples from 125 adults, 26 to 89 years old, with primary gastric adenocarcinoma. Samples were randomly collected during the period of 2005-2013 in Belém city of Pará State, Northern Brazil. Informed consent was obtained prior to sample collection from all adult patients or from the parents or guardians of all juvenile patients. Sample collection was carried out with the approval of the ethics committee of the Human Institute of Health Sciences of the Federal University of Pará (Protocol #35/2010) and João de Barros Barreto University Hospital (Protocol #142004). All patients had negative histories of exposure to chemotherapy and radiotherapy prior to sample collection, and no patient presented with co-occurrence of diagnosed cancers. Data on the clinical features of patients were collected from medical records.

### Histopathology

Endoscopic findings were classified according to the updated Sydney System [20] which considers the degree of inflammation, activity, atrophy and intestinal metaplasia. For each patient, 5 biopsies of gastric tissues were evaluated: 2 from the antral region of the stomach, 1 from the *incisura angularis*, and 2 from the oxyntic mucosa. Chronic gastritis was designated as mild, moderate or severe.

Gastric tumors were classified according to the Lauren classification [21] and staged using standard criteria by pTNM staging [22]. Tables 1 and 2 show the clinicopathological features of gastritis and GC samples, respectively.

**Table 1:** **Clinicopathological features,** ***H. pylori*** **and EBV infection in gastritis samples of juvenile patients**

Variable	***H. pylori***			***CagA***			EBV		
	Negative	Positive	***p***-value	Negative ^c^	Positive	***p***-value	Negative	Positive	***p-***value
**Age (years, Mean ± SD)**	7.45 ± 3.88	12.19 ± 4.09	<0.001*	8.88 ± 4.49	12.45 ± 4.01	0.005*	9.96 ± 4.5	17 ± 1.41	0.033*
**Gender [N(%)]**									
Female	14 (42.4)	19 (57.6)	0.685^a^	23 (69.7)	10 (30.3)	0.096^a^	32 (97.0)	1 (3.0)	0.626^a^
Male	8 (40.0)	12 (60.0)		10 (50.0)	10 (50.0)		19 (95.0)	1 (5.0)	
**Histological subtype [N(%)]**									
Mild	16 (66.7)	8 (33.3)	0.026*^b^	20 (83.3)	4 (16.7)	0.108^b^	23 (95.8)	1 (4.2)	0.136^b^
Moderate/Severe	6 (20.7)	23 (79.3)		13 (44.8)	16 (55.2)		28 (96.6)	1 (3.4)	
**EBV infection [N(%)]**									
Absent	21 (41.2)	30 (58.8)	0.242^b^	31 (60.8)	20 (39.2)	0.998^b^			
Present	1 (50)	1 (50)		2 (100)	0 (0)				

*Significant difference between groups, p < 0.05. ^a^p value after adjustment for age; ^b^p value after adjustment for age and gender; ^c^Negative samples for *H. pylori* and samples with *H. pylori* infection but without CagA virulence factor; EBV: *Epstein-Barr virus*; SD: standard deviation.

**Table 2:** **Clinicopathological features,** ***H. pylori*** **and EBV infection in gastritis samples of adults patients**

Variable	***H. pylori***			***CagA***			EBV		
	Negative	Positive	***p***-value	Negative ^c^	Positive	***p***-value	Negative	Positive	***p***-value
**Age (Mean ± SD)**	35.58 ± 7.82	45.00 ± 10.89	0.011*	37.6 ± 7.67	44.92 ± 11.75	0.039*	42.24 ± 11.14	39.50 ± 3.54	0.733
**Gender [N(%)]**									
Female	3 (21.4)	11 (78.6)	0.410^a^	5 (35.7)	9 (64.3)	0.915^a^	14 (100)	0 (0)	0.999^a^
Male	9 (36.0)	16 (64.0)		10 (40.0)	15 (60.0)		23 (92.0)	2 (8.0)	
**Histological subtype [N(%)]**									
Mild	7 (36.8)	12 (63.2)	0.715^b^	9 (47.4)	10 (52.6)	0.820^b^	17 (89.5)	2 (10.5)	0.999^b^
Moderate/Severe	5 (25.0)	15 (75.0)		6 (30.0)	14 (70.0)		20 (100)	0 (0)	
**EBV infection [N(%)]**									
Absent	12 (32.4)	25 (67.6)	0.999^b^	14 (37.8)	23 (62.2)	0.810^b^			
Present	0 (0)	2 (100)		1 (50.0)	1 (50.0)				

*Significant difference between groups, p < 0.05. ^a^p value after adjustment for age; ^b^p value after adjustment for age and gender; ^c^Negative samples for *H. pylori* and samples with *H. pylori* infection but without CagA virulence factor; EBV: *Epstein-Barr virus*; SD: standard deviation.

### HP and *CagA*detection

The presence of HP was detected by a commercially available rapid urease test (Promedical, Brazil), and the negative results were confirmed by PCR using the oligonucleotides described by Covacci *et al.* [23]. All gastric samples were placed in a tube containing 2% Christensen’s urea agar and examined for urea hydrolysis after 24 h of incubation at 37°C. In the presence of urease produced by HP, the urea is converted to ammonia, resulting in a change of pH and, consequently, the color of the solution.

The detection of the *CagA* gene was carried out by PCR in the gastric mucosa of all patients, using the oligonucleotides described by Covacci *et al.* [23]. All reactions were performed in duplicate. A sample was considered positive if a visible and clear band was observed on a 2% agarose electrophoresis gel.

### EBV detection

EBV was detected by RNA in situ hybridization (ISH) with a 30-bp biotinylated probe (5’-AGACACCGTCCTCACCACCCGGGACTTGTA-3’) complementary to EBV-encoded small RNA-1 (*Eber1*), the most abundant viral product in latently infected cells [24]. Signal amplification was achieved with a mouse anti-biotin antibody (clone BK, 1:20 dilution; DakoCytomation®, CA, USA) and biotinylated rabbit anti-immunoglobulin antibody (polyclonal, 1:100 dilution; DakoCytomation®, CA, USA). The reaction was detected with streptavidin-biotin peroxidase complex (DakoCytomation®, CA, USA) and diaminobenzidine chromogen (DakoCytomation®, CA, USA). The slides were counterstained with Harris’s hematoxylin. Cell analysis was performed by 2 independent investigators using light microscopy, at 40x or 20x magnification. A total of 10 representative microscopic fields were evaluated, and fields containing less than 5 cells were not considered. A gastric cancer sample positive for EBV was included as a positive control, and two slides treated without probe were used as negative controls. Samples where 5% or more of the epithelial cells contained brown/red staining were considered positive. Although lymphocytes were also found to be infected by EBV, we did not include infected lymphoid cells in our analysis.

### Statistical analyses

The Shapiro-Wilk test was used to evaluate the distribution of age data and to determine the appropriate subsequent test for statistical comparison. The Mann-Whitney test (non-parametric) or T-test for independent samples (parametric) was used to compare ages between the groups. Associations between HP or EBV and other clinicopathological features were analyzed using chi-square (χ^2^) and logistic regression. A p-value less than 0.05 was considered significant, and the confidence interval was 95%.

## Results

We investigated 226 individuals, including 92 women and 134 men, divided into three groups. The percentage of men was 38.7%, 64.1% and 68% for juvenile UE patients, adult UE patients and GC patients, respectively. The proportion of males was higher in the cohort of GC patients (p < 0.001, OR = 3.365, 95% CI = 1.784 – 6.345) and adult UE patients (p = 0.014, OR = 2.827, 95% CI = 1.233 – 6.485) than among juvenile UE patients.

Among juvenile UE patients, 40 patients presented gastritis by UE. However, 59% of patients without UE-diagnosed gastritis presented mild gastritis by histopathological analysis. Therefore, 53 (85.5%) patients showed some degree of gastritis in the group of juvenile UE patients. The age of patients without gastritis did not differ from that of patients with gastritis [median ± interquartile range (IQR): 7.33 ± 8 vs 10.23 ± 8 years old; p = 0.080, Mann-Whitney test]. However, patients with mild gastritis were younger than patients with moderate or severe gastritis (mean ± standard deviation (SD): 8.25 ± 4.30.90 *vs* 11.86 ± 4.27 years old; p = 0.004, T-test). The gender breakdown did not differ between juvenile patients with and without gastritis (p = 0.725), as well as between juvenile patients with mild gastritis and moderate or severe gastritis (p = 0.097).

**Figure 1:**
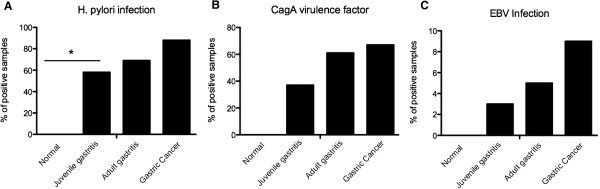
**Pathogen frequency in normal gastric mucosa, gastritis of juveniles, gastritis of adults and gastric cancer samples. A)** *H. pylori* infection. **B)** *CagA* virulence factor of HP. **C)** EBV infection. *Significant difference between groups by logistic regression, after adjustment for age and gender (p < 0.05).

Among adult UE patients, all of the evaluated individuals presented gastritis, including 19 (48.7%) with mild gastritis and 20 (51.3%) with moderate or severe gastritis. Patients with mild gastritis were younger than patients with moderate or severe gastritis (mean ± SD: 37.47 ± 7.20 vs 46.50 ± 12.07 years old; p = 0.003, T-test). The gender breakdown did not differ between juveniles patients with mild gastritis and moderate or severe gastritis (p = 1) in this group of analysis.HP infection was detected in 0% of normal gastric mucosa samples, 58.5% of samples from juvenile gastritis patients, 69.2% of adult gastritis samples and 88% of GC patients (Figure 1a). In juvenile individuals, HP infection was more frequently observed in gastritis samples than in normal samples (p = 0.004, Yates correction). The frequency of HP in adult gastritis did not differ from the observed frequency of gastritis in juvenile patients (p = 1.000, after adjustment for age and gender) or in the GC samples (p = 0.335, after adjustment for age and gender).

HP *CagA*^*+*^ was detected in 0% of normal gastric mucosa samples, 37.7% of samples from juvenile gastritis patients, 61.5% of adult gastritis samples and 67.2% of GC patients (Figure 1b). The frequency of infection by HP *CagA*^*+*^ did not differ between samples from juvenile patients with gastritis and normal gastric mucosa (p = 0.064, Yates correcton). Moreover, the frequency of HP *CagA*^*+*^ in adult gastritis tissue samples did not differ from that observed in juvenile gastritis patients (p = 1, after adjustment for age and gender) or in GC samples (p = 0.500, after adjustment for age and gender).

The frequency of HP infection did not differ between males and females in the samples from juvenile or adult patients evaluated by UE (p > 0.05, after adjustment for age; Tables 1 and 2). However, in GC samples, HP infection was detected more frequently in males than in females (p = 0.023 OR = 3.651, 95% CI = 1.190 – 11.199, after adjustment for age; Table 3).

**Table 3:** **Clinicopathological features,** ***H. pylori*** **and EBV infection in gastric tumors**

Variable	***H. pylori***			***CagA***			EBV		
	Negative	Positive	***p***-value	Negative ^c^	Positive	***p***-value	Negative	Positive	***p***-value
**Age (Median ± IQR)**	57 ± 25	64 ± 16.75	0.027*	57 ± 22	64.5 ± 16.5	0.027*	63 ± 20	67 ± 21.5	0.192
**Gender [N(%)]**									
Female	9 (22.5)	31 (77.5)	0.023*^a^	15 (37.5)	25 (62.5)	0.307^a^	39 (97.5)	1 (2.5)	0.115^a^
Male	6 (7.1)	79 (92.9)		26 (30.6)	59 (69.4)		74 (87.1)	11 (12.9)	
**Tumor location [N(%)]**									
Non-cardia	12 (16.4)	61 (83.6)	0.080^b^	26 (35.6)	47 (64.4)	0.519^b^	67 (91.8)	6 (8.2)	0.592^b^
Cardia	3 (5.8)	49 (94.2)		15 (28.8)	37 (71.2)		46 (88.5)	6 (11.5)	
**Histological subtype [N(%)]**									
Intestinal-type	6 (8.5)	65 (91.5)	0.247^b^	25 (46.3)	29 (53.7)	0.012*^b^	63(88.7)	8 (11.3)	0.650^b^
Diffuse-type	9 (16.7)	45 (83.3)		16 (22.5)	55 (77.5)		50 (92.6	4 (7.4)	
**Stage [N(%)]**									
Early	7 (15.9)	37 (84.1)	0.680^b^	25 (56.8)	19 (43.2)	0.000*^b^	41 (93.2)	3 (6.8)	0.999^b^
Advanced	6 (8.3)	66 (91.7)		14 (19.4)	58 (80.6)		64 (88.9)	8 (11.1)	
**Tumor invasion [N(%)]**									
T1/T2	4 (13.3)	26 (86.7)	0.453^b^	13 (43.3)	17 (56.7)	0.616^b^	29 (96.7)	1 (3.3)	0.560^b^
T3/T4	11 (11.6)	84 (88.4)		28 (29.5)	67 (70.5)		84 (88.4)	11 (11.6)	
**Lymph node metastasis [N(%)]**									
Absent	3 (25)	9 (75.0)	0.193^b^	8 (66.7)	4 (33.3)	0.024*^b^	12 (100)	0(0)	0.999^b^
Present	12 (10.6)	101 (89.4)		33 (29.2)	80 (70.8)		101 (89.4)	12 (10.6)	
**Distant metastasis [N(%)]**									
Absent	11 (16.7)	55 (83.3)	0.136^b^	30 (45.5)	36 (54.5)	0.005*^b^	62 (93.9)	4 (6.1)	0.258^b^
Present	4 (6.8)	55 (93.2)		11 (18.6)	48 (81.4)		51 (86.4)	8 (13.6)	
**EBV infection [N(%)]**									
Absent	15 (13.3)	98 (86.7)	0.998^b^	39 (34.5)	74 (65.5)	0.358^b^			
Present	0 (0)	12 (100)		2 (16.7)	10 (83.3)				

*Significant difference between groups, *p* < 0.05. ^a^p value after adjustment for age; ^b^p value after adjustment for age and gender; ^c^Negative samples for *H. pylori* and samples with *H. pylori* infection but without CagA virulence factor; EBV: *Epstein-Barr virus*; IQR: interquartile range.

In juvenile patients who underwent UE, gastritis patients with HP infection and with HP *CagA*^*+*^ were older than those without this pathogen (p < 0.001 and p = 0.005, respectively, T-test; Table 1). In this group of patients, HP infection was more prevalent in cases of moderate or severe gastritis than in those of mild gastritis (p = 0.026; OR = 5.136, 95% CI = 1.220 – 21.611, after adjustment for age and gender; Table 1). As observed in juvenile patients, adults with gastritis who were also positive for HP infection and HP *CagA*^*+*^ were older than those without this pathogen (p = 0.011 and p = 0.039, respectively, T-test; Table 2).

In tumor samples, patients with HP *CagA*^*+*^ were older than patients without HP *CagA*^*-*^ (p = 0.027, Mann-Whitney test; Table 3). HP *CagA*^*+*^ was more prevalent in intestinal-type than diffuse-type GC (p = 0.012; OR = 2.741, 95% CI 1.252 – 6.001, after adjustment for age and gender; Table 3). The presence of HP *CagA +* was also associated with lymph node metastasis (p = 0.024; OR = 5.611, 95% CI = 1.255 – 25.097, after adjustment for age and gender) and distal metastasis (p = 0.005; OR 3.299, 95% CI = 1.441 – 7.556, after adjustment for age and gender; Table 3).

**Figure 2:**
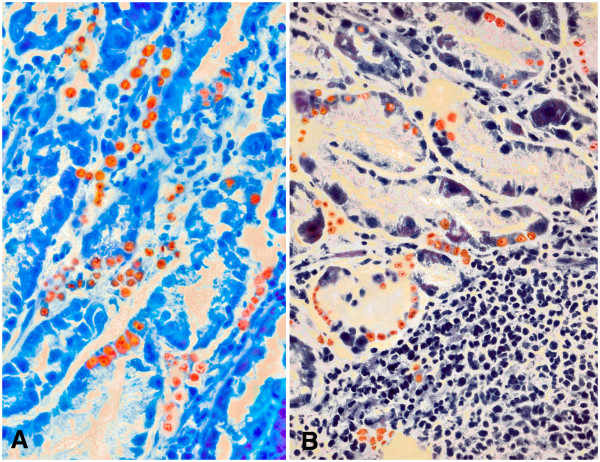
**Detection of EBV by** ***in situ*** **hybridization.** Strong nuclear staining was observed in infected nuclei in a **A)** mild gastritis (40x) and **B)** adult moderate gastritis.

EBV infection was detected in 0% of normal gastric mucosa samples, 3.8% of samples from juvenile gastritis patients, 5.1% of samples from adult gastritis patients and 9.6% of GC patients (Figure 1c and Figure 2). In the gastric mucosa, we found that 5-15% of cells were infected. Rates of EBV infection did not differ among normal gastric mucosa and mucosa from juvenile patients with gastritis (p > 0.05, Yates correction). Moreover, the frequency of EBV infection in the gastritis of adults did not differ from that observed in the gastritis of juvenile patients and GC samples (p > 0.05, after adjustment for age and gender). No association between EBV infection and HP infection or any clinicopathological variable was found (p > 0.05, Table 1, 2 and 3).

Although we did not observe a statistically significant association between the two pathogens, only one EBV-positive case was found without concomitant HP infection. This case was an 18-year-old female with no sign of gastric disease by UE evaluation and mild gastritis by histopathological analysis.

## Discussion

Infection by HP and EBV occurs most often during childhood, and both viruses can synergistically enhance the alteration of gastric mucosa to chronic gastritis and GC [6, 17, 19, 25].

Gastritis is more likely to occur in older adults but can affect people of all ages, including children. Many studies [26, 27] have attempted to understand the development of gastritis in children. Souza *et al.* [28], identified endoscopic abnormalities in 74% of the children and adolescents studied. Among these, 26% (7/21) had gastritis as determined by UE. In our study, gastritis was identified by UE in a larger number of juvenile patients (40/62). However, finding an apparently normal mucosa by endoscopy does not exclude the possibility of pathological change, as biopsy is required for a definitive determination. Our results confirm this assertion because in 22 normal endoscopic exams, only 9 showed a normal histological pattern. In addition, patients with mild gastritis were younger than patients with moderate or severe gastritis, perhaps because they had not yet been exposed to many aggressive agents that can lead to gastritis [6].

In the present study, HP infection was detected in most of the UE patients and GC samples, although the techniques used may underestimate the presence of the bacteria. Other studies in Brazil, such as those by Gatti *et al*. [29] and Souza *et al.* [28], reported the prevalence of HP infection in juveniles to be 51% and 60%, respectively. These results are consistent with our findings. An investigation in other developing countries also showed a similar frequency (61.8%) [30]. In adults, a slightly higher frequency was found in other studies in Brazilian (88.4%) [31] and African (70–97%) populations [32]. These numbers most likely reflect the social and health conditions of the studied populations because infections by HP are more common in developing countries than in developed countries [19].

In our study, HP infection was more common in cases of moderate or severe gastritis than in juveniles with mild gastritis. Similarly, Álvarez *et al.* [31] found a higher frequency of moderate and severe gastritis in patients infected by HP. Additionally, we observed that the prevalence of HP, particularly HP *CagA*^*+*^, increased with age, corroborating previous investigations in populations from northeastern Brazil, China, and Japan [31–33]. It has been suggested that the earlier HP infection occurs, the greater the risk for GC due to chronic inflammatory reactions to the infection [34]. In the population studied here, the frequency of infection by HP or HP *CagA*^*+*^ in GC patients did not differ from that observed in adults patients with gastritis. However, some studies have found that the spontaneous disappearance of HP during malignant transformation of gastric epithelia is possible [35, 36]. Nevertheless, cancer still occurs after successful eradication of HP; therefore, eradication of HP does not lead to a significant decrease in the incidence of gastric cancer [37]. Furthermore, it has been observed that the eradication of HP must occur before carcinomatous change develops [38]. This finding highlights the necessity of epidemiological studies to understand the incidence and prevalence of HP in a population and to help in the development of population-specific strategies to prevent and control HP.

The prevalence of HP in gastric tumors varies with the country being analyzed [39]. In Brazil, a previous study detected this bacteria in 85.7% of gastric tumor samples [40], which is similar to the frequency observed in our study (88%). In addition, we observed that the frequency of HP was 1.5-times greater in GC samples than in juvenile gastritis samples, and almost ninety times higher than in normal gastric mucosa, highlighting a strong association between HP and the process of gastric carcinogenesis. After initial infection by HP, patients develop acute gastritis. This may resolve spontaneously, but the majority of cases progress to chronic gastritis [41]. The clinical outcome of HP infection is determined by the complex interaction between host factors and bacteria [42]. *CagA* is likely the most significant virulence factor [43] and is strongly associated with the risk for GC [42]. It is known that the basic *CagA* genotype acquired in childhood remains throughout life [44].

HP *CagA*^*+*^ strains have been associated with more intense inflammation and greater bacterial density, as well as progression to gastric atrophy, peptic ulcer and gastric cancer [43]. However, the involvement of HP *CagA*^*+*^ in gastric carcinogenesis in Brazilian individuals is still controversial. Oliveira *et al.* [45] found an association between the presence of *CagA* with more marked antral inflammation in duodenal ulcers (90%) and gastric carcinoma (94.23%) in Brazilian adults. In another study of the Brazilian population, Gatti *et al*. [29] found a slightly greater frequency (69%) of *CagA*^*+*^ in patients with chronic gastritis. However, the authors did not find any association between *CagA*^*+*^ strains and chronic gastritis, suggesting that other bacterial factors are involved in disease genesis. Consistent with this, in our study we did not find an association between HP CagA^+^ and the presence of gastritis. However, HP CagA^+^ was associated with poor prognostic variables in GC cancer.

Here, the *CagA*^*+*^ genotype was associated with the age, histological subtype and metastatic process of GC patients. Unlike Kuo *et al.* [46] *,* we found a higher frequency of *CagA*^*+*^ patients in the older cohort of our Brazilian population. Moreover, the presence of HP *CagA*^*+*^ was higher in intestinal-type than in diffuse-type GC. The transformation in gastric mucosa induced by HP *CagA*^*+*^ is similar to the alterations that occur in the intestinal-type of GC [2, 47]; thus, finding the highest frequency (84.6%) of *CagA*^*+*^ in intestinal-type GC is reasonable. Additionally, the presence of HP *CagA*^*+*^ may induce tumor alterations that lead to metastasis. Kong *et al.* [48] observed that HP stimulates the synthesis of CACUL1 in a gastric tumor cell line, which in turn promotes the expression of matrix metalloproteinase 9 and increases invasion and metastasis.

Approximately 7% of our patients presented EBV infection in gastric tissue samples. None of these patients presented symptoms of mononucleosis. Therefore, EBV infection in gastric mucosa is not necessarily associated with infection in lymph nodes or tonsils.

The frequency of infection by EBV in GC (9.6%) was similar to the frequency previously described in other populations (approximately 7.3 – 13%) [49–51]. However, the prevalence of EBV infection in patients with gastritis (3.8% among juvenile and 5.1% among adults) was reduced in comparison to the prevalence reported in the literature. Ryan *et al*. [25] found EBV sequences in 83% (5/6) of adult and 30% (15/50) of pediatric gastritis lesions. The low frequency identified in gastric mucosa of the younger patients may be because the stomach is not the primary location for B lymphocytes immortalized by EBV infection. The gastric mucosa does not seem to possess the necessary homing mechanism for settlement by these infected lymphocytes [5].

Although our results suggest that EBV is not critical for the initial steps of gastric mucosal injury in the Brazilian population, the presence of EBV genomes and their expression in gastric carcinoma cells raises the possibility that this virus may contribute to neoplastic transformation. Notably, EBV was found within the malignant cells in approximately 10% of gastric adenocarcinomas, and this infection seems to precede malignant transformation [52]. Additionally, a previous *in vitro* study reported that reactive products from HP infection trigger EBV reactivation in latently infected gastric epithelial cells [53]. Inflammatory stress that occurs during the malignization of gastric cells may lead to infiltration of lymphocytes carrying EBV. This process may increase the possibility of contact with epithelial cells, or it may produce a rich medium that supports the cytokine-stimulated clonal replication of EBV-infected epithelial cells [16].

Recently, Cárdenas-Mondragón [54] reported that co-infection with EBV and HP *CagA*^*+*^ was associated with the presence of severe gastritis, reveling a critical role for EBV in gastric mucosal alterations. Here, we did not find an association between EBV and HP or the presence of *CagA* in our gastritis or cancer samples, most likely due to the low frequency of EBV.

The main limitation of this study is its relatively small sample size. Therefore, some of the statistical analyses presented reduced the power to detect significant differences between groups. Therefore, further investigations are still necessary to fully understand the roles of EBV and HP in gastric carcinogenesis in the Brazilian population.

## Conclusion

Our results strongly suggest that HP is involved in the pathophysiology of severe gastric lesions and development of GC in infected juveniles and adults. Furthermore, the frequency of these pathologies increases with age. HP *CagA*^*+*^ was associated with greater inflammation and more advanced stage of cancer. EBV was not the primary pathogenic factor in our sample. Additionally, a better understanding of the most prevalent strains of pathogens, and their associated antigens, will be valuable for the development of vaccines clinical protocols for screening and treating infection.

## Authors’ information

CRTS: Biomedical research with PhD in Genetic and Molecular Biology, experience in Human Genetics with an emphasis on carcinogenesis and molecular markers, as well as experience in diagnostic imaging; KSO: professor at School of Medicine - UFPA; JJSF: pharmaceutical assistant professor at University Center of Pará (CESUPA) with a PhD in Cell Biology; MFL: researcher at the Department of Orthopedics and Traumatology and at the Department of Morphology and Genetics -UNIFESP, with experience in genetics and gastric cancer; DQC: visiting professor at the Center for Research in Oncology-UFPA, with experience in genetics and gastric cancer; ADS: PhD student in Genetics and Molecular Biology at UFPA; ASK: associate professor at the Institute of Biological Sciences and Center for Research in Oncology-UFPA, with experience in genetics with an emphasis on Human and Medical Genetics; RCM: assistant professor at UFPA, experience in pharmacology with an emphasis on oncology; APNNA: pathologist and professor at UFC; PPA: surgeon at University Hospital João de Barros Barreto-UFPA, experience in oncology and gastroenterology; MCS: Full professor of genetics, Head of Genetics Division, experience in Human Genetics and cancer; RRB: associate professor at the Institute of Biological Sciences-UFPA, experience in Human Genetics with an emphasis on carcinogenesis and mutagenesis.

## Abbreviations

HP: *Helicobacter pylori*
EBV: *Epstein-Barr virus*
UE: Upper endoscopy
GC: Gastric cancer
IARC: International agency for research on cancer
*CagA*: Cytotoxin associated gene A.
